# Differences in prevalence of self-reported musculoskeletal symptoms among computer and non-computer users in a Nigerian population: a cross-sectional study

**DOI:** 10.1186/1471-2474-11-177

**Published:** 2010-08-06

**Authors:** O Ayanniyi, BOO Ukpai, AF Adeniyi

**Affiliations:** 1 Department of Physiotherapy, College of Medicine, University of Ibadan, Ibadan, Nigeria; 2 Department of Physiotherapy, Ladoke Akintola University of Technology Teaching Hospital, Osogbo, Nigeria

## Abstract

**Background:**

Literature abounds on the prevalent nature of Self Reported Musculoskeletal Symptoms (SRMS) among computer users, but studies that actually compared this with non computer users are meagre thereby reducing the strength of the evidence. This study compared the prevalence of SRMS between computer and non computer users and assessed the risk factors associated with SRMS.

**Methods:**

A total of 472 participants comprising equal numbers of age and sex matched computer and non computer users were assessed for the presence of SRMS. Information concerning musculoskeletal symptoms and discomforts from the neck, shoulders, upper back, elbows, wrists/hands, low back, hips/thighs, knees and ankles/feet were obtained using the Standardized Nordic questionnaire.

**Results:**

The prevalence of SRMS was significantly higher in the computer users than the non computer users both over the past 7 days (χ^2 ^= 39.11, p = 0.001) and during the past 12 month durations (χ^2 ^= 53.56, p = 0.001). The odds of reporting musculoskeletal symptoms was least for participants above the age of 40 years (OR = 0.42, 95% CI = 0.31-0.64 over the past 7 days and OR = 0.61; 95% CI = 0.47-0.77 during the past 12 months) and also reduced in female participants. Increasing daily hours and accumulated years of computer use and tasks of data processing and designs/graphics significantly (p < 0.05) increased the risk of reporting musculoskeletal symptoms. Over the past 7 day duration, the neck (33.9%) and low back (11.4%) had highest prevalence of SRMS for the computer and non computer users respectively.

**Conclusion:**

The prevalence of SRMS was significantly higher in the computer users than the non computer users and younger age, being male, working longer hours daily, increasing years of computer use, data entry tasks and computer designs/graphics were the significant risk factors for reporting musculoskeletal symptoms among the computer users. Computer use may explain the increase in prevalence of SRMS among the computer users.

## Background

The availability of computers have made varieties of work faster, easier, neater and less frustrating to the users. Computers (also called video display terminals) have become increasingly common in both workplaces and homes over the past 20 years [1]. However, the use of computers is not without its menacing side; an example of which is the predisposition to musculoskeletal disorders by the users. Sustained pain in the upper extremity and neck regions and specific musculoskeletal disorders, such as wrist tendonitis, epicondylitis, and trapezius muscle strain are high among computer users [2]. Non-specific forearm pain has been reported as a common complaint among computer workers with high job demands, time pressure, and female gender being some of the risk factors [3]. More reasons have been adduced for musculoskeletal problems in computer users. Frequent computer-related activities have also been shown to be an independent risk factor for neck, shoulder and low back pain and independently related to the intensive use of the mouse device and to a lesser extent to keyboard usage [4]. In adolescents according to Hakala et al [4], daily use of computers exceeding 2-3 hours seems to be a threshold for neck shoulder pain and exceeding 5 hours for low back pain. In view of the widespread use of the computer, even relatively small risks associated with their use would have important public health implications, and interventions that result in decreased risk would have great public health benefits [1].

Different prevalence statistics on the SRMS experienced by computer users have been documented for different anatomical parts of the body. The seven days prevalence of moderate to severe forearm pain in computer users as documented by Kryger et al [3] was 4.3% while one year incidence of reported symptom cases was 1.3%. The prevalence of weekly severe pain was low, the incidence of prolonged neck pain was 0.31%, and the incidence of prolonged shoulder pain was 0.23% [5]. The baseline prevalence of hand/wrist or elbow pain was 24% (moderate pain in last 7 days) and after one year new cases arose in 7.7% of the population [6]. Andersen et al [5] reported the prevalence of possible carpal tunnel syndrome as 1.4-4.8% and the incidence of new cases in 12 months as 5.5% based on symptoms, and 1.2% when confirmed by a clinical interview. According to Hakala et al [4], computer-related activities may explain the increase of neck shoulder pain and low back pain presented in the 1990s and the beginning of 2000 in the Finnish population. In a two year study of 3475 computer users in Denmark, self-reported use of a computer more than 75% time, compared with 50% of time, increased the risk of hand/wrist symptoms, while using a mouse for 50% of time, compared with 25% of time, increased risk for female users [7]. A systematic review also concluded that there is consistent evidence of a positive relationship across numerous prospective and cross-sectional studies with increased risk of disorders most pronounced beyond 20 hours/week of computer use or with increasing years of computer work [8].

However, most of the prevalence reports on SRMS and computer use have been conflicting with results as varied as the number of studies carried out in this area. A more recent study for instance has questioned the beliefs about the prevalence of adverse effects from the use of computers. Andersen et al [9] reported that the common opinion stating that computer use has adverse health effects is questionable. According to the authors, most computer workers have minor or no neck and shoulder pain complaints, few experience prolonged pain, and even fewer, chronic neck and shoulder pain. Despite the increasing number of computer users and the abundance of evidence suggesting that SRMS are present in computer users, there are very few readily available and conclusive studies that have compared these prevalence with that of the non computer users. Most previous studies failed to establish whether the prevalence was peculiar to computer users. In view of this, it is difficult stating whether the prevalence of SRMS in computer users is the same or different from non computer users. This study was hence designed to find if any difference existed in the prevalence of SRMS between the computer and non computer users and to investigate the risk factors associated with SRMS among the computer users.

## Methods

### Study design

This was a cross sectional study of computer and non computer users.

### Recruitment of study population

A total of 300 questionnaires were distributed to computer users based on a calculated representative sample size of 295.65 [10]. This calculation considered standard normal deviate (1.96), proportion in the target population estimated to have musculoskeletal symptoms (74% based on an unpublished data by Dada, 2004) and degree of accuracy of 0.05 alpha levels. Out of the 300 questionnaires distributed, 236 were returned yielding a response rate of 78.1%. Subsequently, the questionnaires were administered on 236 age- and sex-matched non computer workers who consented to participate in the survey giving a total of 472 participants in the two groups. The computer users were recruited from the Ladoke Akintola University of Technology Teaching Hospital, Osogbo; College of Health Sciences, Osogbo; Power Holdings Company of Nigeria, Osogbo; Bureau of Computer Services and Information Management, Osogbo and computer operators in private computer business centres in Osogbo all in Osun State, South-Western Nigeria. For this study, the computer users were the workers whose employment were anchored essentially on the use of the computer, while the non computer users who served as the control group were individuals who only used the computer casually for activities like e-mailings and for less than 30 minutes in a week. They were members of staff of the five centres from where the computer users were recruited and they were recruited purposively into the study.

### Data collection procedure

A letter of transmission explaining the purpose and nature of the study was attached to the questionnaire. This also contained adequate instructions on how to fill the questionnaire. The SRMS were assessed using the self administered Standardized Nordic questionnaire developed by Kuorinka et al [11]. All the participants were able to read and understand the questionnaire in English language; hence we did not have to subject the instrument to translations into local languages. The participants were queried concerning musculoskeletal symptoms on the neck, shoulders, upper back, elbows, wrists/hands, low back, hips/thighs, knees and ankles/feet in the form of musculoskeletal symptoms (troubles, aches or pain) felt over the past 7 days and during the past 12 months prior to the study. The choice of the time frames was predetermined and was adapted from a previous study [3]. This presented the opportunity for the documentation of the current (short term) and past (long term) SRMS. All the participants were asked questions such as "Have you at any time during the past 12 months had trouble (ache, pain, discomfort) in your neck, shoulders, elbows etc?" They were also asked whether they had any troubles in the over the past 7 days. In addition, they were asked to indicate using a diagram on what part of the body the pain, ache or discomfort was being felt. The SRMS were present if a respondent answered "yes" to the questions and ruled out if the respondent answered "no". The self reported estimated time duration during for which computer was used by each participant on daily basis and the accumulated years of computer use were also assessed. The participants were also asked to respond to the question probing most of the task they carried out with their computers. Further information was also documented on the demographic characteristics of the participants. The questionnaire was administered on the participants following an approval granted by the joint University of Ibadan and University College Hospital Ethical Review Committee. The consent of the appropriate officers in charge of each of the establishments where the study took place as well as that of the participants was also obtained.

### Statistical analysis

The differences in the prevalence of SRMS between the computer and non computer users over the past 7 days and during the past 12 months were compared using the Pearson Chi square test. The prevalence is presented in the form of percentage and frequency distributions. The prevalence ratio was calculated using a 2 by 2 table, as the ratio of the prevalence of SRMS in the computer user group divided by the prevalence of SRMS in the non computer user group. A regression analysis was also carried out to investigate significant risk factors for reporting musculoskeletal symptoms among the computer users. A predictive equation was developed using multiple logistic regression analysis of data collected from the 236 computer users. The equation incorporated age, sex, daily hours of computer use, total years of computer use and computer task as independent covariates for prediction of SRMS. Data were analysed using the SPSS Version 15 (SPSS Inc, Chicago, IL, U.S.A.).

## Results

### Self reported musculoskeletal symptoms among the computer and non computer users

**Figure 1:**
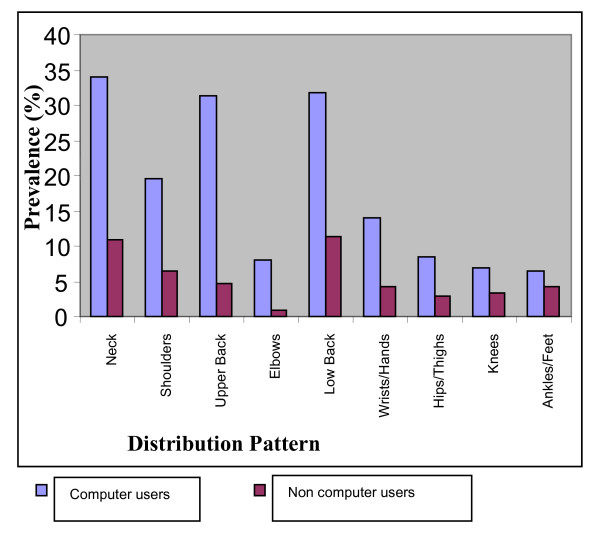
**Seven day prevalence of SRMS among computer and Non computer users by anatomical distribution**.

**Figure 2:**
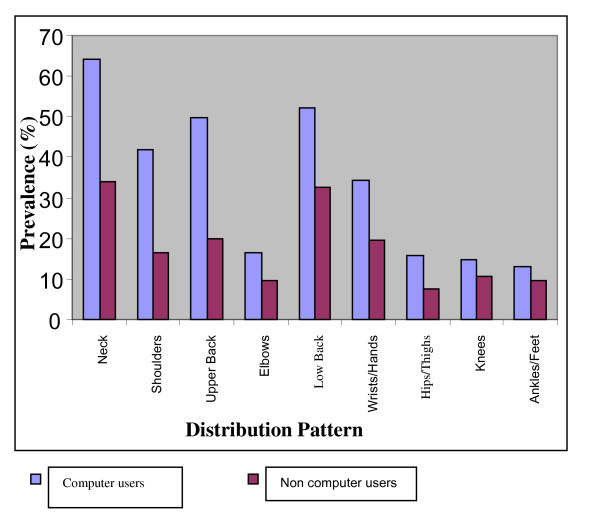
**Twelve month prevalence of SRMS among computer and non computer users by anatomical distribution**.

The mean age of the participants were 29 ± 4.87 (for computer users) and 31 ± 6.23 (for non computer users) and most of them were males (57.6%) (Table 1). Table 1 also shows a significant difference (p = 0.001) in the amount of time spent daily on the computer by the two groups. Comparing the prevalence of SRMS between the computer and non-computer users over the past 7 days revealed a significantly higher prevalence of SRMS in the computer users than the non computer users (*χ*^2 ^= 39.11, p = 0.001, prevalence ratio 2.03) as seen in table 2. The difference in the prevalence of SRMS during the past 12 months between the computer and the non computer users was significant as well (*χ*^2 ^= 53.56, p = 0.001, prevalence ratio 2.7) with higher prevalence in the computer users. In terms of the anatomical distribution of SRMS over the past 7 day duration, the neck was mostly reported as the site of SRMS in the computer users (33.9%) while low back was the most prevalent (11.4%) for the non computer users (figure 1). During the past 12 months for both groups (figure 2), the neck was the site with the most prevalent SRMS (64.0% and 33.9% for the computer and non computer users respectively). Over the past 7 days, the ankle/feet were least reported (6.4%) site of MSD in the computer users while the elbow was least reported (3.0%) in the non computer users. During the past 12 months in the computer users, the low back was next in prevalence to the neck and similarly, the least SRMS was reported in the ankles/feet (13.1%). For the non computer users however, hips/thighs were the least reported site of SRMS (7.6%) during the past 12 month duration.

**Table 1:** Distribution of respondents in the computer and non computer user groups

	Computer	Non computer	p value
	Users (n = 236)	users (n = 236)	
Age (years)	29 ± 4.87	31 ± 6.23	0.072
Daily computer duration (mins)	180 ± 17.52	7 ± 2.14	0.001
**Sex**			
Males	136 (57.6%)	136 (57.6%)	
Females	100 (42.4%)	100 (42.4%)	
**Marital status**			
Single	139 (58.9%)	117 (49.6%)	
Married	97 (41.1%)	119 (50.4%)	
**Duration of daily computer use by age range (mins)**			
<20	136 ± 18.22	12 ± 1.86	
21-30	187 ± 31.45	8 ± 3.21	
31-40	97 ± 11.16	12 ± 4.50	
>40	74 ± 14.91	5 ± 1.33	

**Table 2:** Comparison of self reported prevalence of musculoskeletal symptoms among the computer and non computer users

	Computer users	Non computer users	χ^2^	Prevalence
	(n = 236)	(n = 236)		Ratio
**Duration**				
Over the past 7 days	55.9%	27.5%	**39.11	2.03
The past 12 months	93.2%	33.9%	**53.56	2.74

**Significant at p < 0.01

### Factors that contributing to the prevalence of self reported musculoskeletal symptoms among the computer users

There was a clear reduction in the odds of reporting musculoskeletal symptoms with increasing age among the computer users both over the past 7 days and during the past 12 months (Table 3). The odds of reporting musculoskeletal symptoms was least for participants above the age of 40 years for both time frames (OR = 0.42, 95% CI = 0.31-0.64 over the past 7 days) and (OR = 0.61; 95% CI = 0.47-0.77 during the past 12 months). Being female also significantly reduced the odds of reporting musculoskeletal symptoms over the past 7 days and during the past 12 months. Although being married appears to reduce the risk of reporting musculoskeletal symptoms, this was however not significant. As the increase in the estimated daily hours of computer use significantly increased the risk of reporting musculoskeletal symptom among the computer users in both time frames, the possibility of reporting musculoskeletal symptom during the past 12 months also increased as the accumulated years of computer use increased. However, the use of the computer above six years was the only significant risk of reporting musculoskeletal symptom among the computer users over the past 7 days (OR = 1.72; 95% CI = 1.41-2.15). In terms of the computer work tasks, both data entry and designs/graphics increased the odds of reporting musculoskeletal symptoms in both the past 7 days and during the past 12 months however, the task of design/graphics contributed most to the risks of reporting musculoskeletal symptoms (OR = 2.55; 95% CI = 2.04-2.98 over the past 7 days) and (OR = 6.62; 95% CI = 5.16-6.94 during the past 12 months). A predictive equation was obtained as prevalence of SRMS = 11.346 + 0.0146 (age in years) + 3.1278 (male) + 1.6132 (hours per day) + 2.2641 (years of computer use) + 0.0248 (data entry task).

**Table 3:** Odds Ratios for prevalence of self reported musculoskeletal symptoms by demographic and work characteristics

	SRMS		SRMS	
	over the past 7 days		during the past 12 months	
	OR (95% CI)		OR (95% CI)	
**Age**				
<20	1	Reference	1	Reference
21-30	0.83	0.67-0.92*	0.94	0.73-1.58
31-40	0.65	0.58-0.84*	0.74	0.51-0.84*
>40	0.42	0.31-0.64*	0.61	0.47-0.77*
**Sex**				
Male	1	Reference	1	Reference
Female	0.42	0.16-058*	0.74	0.55-0.63*
**Marital Status**				
Single	1	Reference	1	Reference
Married	0.75	0.52-1.36	0.94	1.73-1.47*
**Daily time of computer use**				
<2 hours	1	Reference	1	Reference
2-4 hours	1.36	0.92-1.68*	3.25	1.84-4.73*
>4 hours	4.12	3.21-5.16*	.04	3.66-6.33*
**Accumulated years of computer use**				
<1 year	1	Reference	1	Reference
1-3 years	0.95	0.62-1.35	1.34	1.16-1.82*
4-6 years	1.25	0.73-1.56	2.51	1.88-2.97*
>6 years	1.72	1.41-2.15*	4.29	3.64-5.01*
**Work Task**				
Word processing	1	Reference	1	Reference
Data entry	1.73	1.52-1.95*	2.28	1.79-2.58*
Designs/graphics	2.55	2.04-2.98*	6.62	5.16-6.94*
Others	0.82	0.55-1.48	1.36	0.78-1.74

*Significant at p < 0.05

## Discussion

The main finding from this study shows that the computer users had significantly higher prevalence of SRMS than the non computer users over the past 7 days and during the past 12 months. Opinions have varied on the actual contribution of computer use to the prevalence of SRMS in the users. The fact that many studies [3,4,9,12] among others did not compare the prevalence of SRMS between the computer and non computer users made it difficult to confirm whether or not the use of computer actually added to the prevalence of SRMS. Inconsistent findings in epidemiological studies exploring the relation between the use of computer and forearm pain for instance have led to the controversy as to whether the use of computers increases the risk of arm symptoms and disorders [3]. However, with our finding from the comparison of SRMS prevalence between the computer and non computer users, we are able to say that computer use adds to the possibility of higher SRMS because the prevalence was significantly lower in their age and sex matched individuals who were not computer users. The higher prevalence of SRMS in the computer users may result from one or combination of factors including posture, work tasks, furniture and positioning of the computers relative to the users. According to Eltayeb et al [13] awkward head and body posture, irregular body posture and task difficulty, number of working hours with the computer were significantly associated with neck complaints among computer users.

Assessments across the study periods revealed that the computer users reported lesser musculoskeletal symptoms over the past 7 days than during the past 12 months. The lesser prevalence of SRMS observed in the 7 day duration could be because fewer of the computer users were experiencing the symptoms of musculoskeletal disorders within the week that the questionnaires were filled. Conversely the higher prevalence of SRMS of about ninety out of a hundred computer users during the past 12 month could be indicating that most of them had reported one musculoskeletal symptom or the other during the last 12 months.

It is worth reporting that over the past 7 days and during the last 12 month durations, the non computer users also reported musculoskeletal symptoms. However, the prevalence was much lower than that of the computer users for the respective periods. This indicated that musculoskeletal symptoms are reported by many individuals regardless of whether they used the computer or not but the use of the computer could have contributed to the increase in prevalence seen in the computer users especially for some body parts. The prevalence ratio of more than one for the computer users to the non computer users in both assessment periods indicates the possibility of the computer use contributing to the prevalence of the SRMS in both the short and the long terms. This piece of information will help to further strengthen the fact that computer use actually contributes to the possibility of having higher prevalence of SRMS. Rempel et al [2] reported that sustained pain in the upper extremity and neck regions and specific musculoskeletal disorders, such as wrist tendonitis, epicondylitis, and trapezius muscle strain were reported as high among computer users.

Considering the anatomical distribution of SRMS over the past 7 days only, computer users had most of their SRMS around the neck while in the non computer users it was at the low back. The low back was the area with the highest prevalence of SRMS in the non computer users because it is where many individuals traditionally report discomfort regardless of their job demands. The structures around the ankles were usually relaxed and not put into active use by the computer users and that may account for why SRMS was least in this part of the body. However both the computer and non computer users had their highest prevalence of SRMS at the neck region during the past 12 month duration. More musculoskeletal pain during computer work was reported to be easily felt in the neck and shoulder areas than in the lower back [4]. This may be expected especially in the computer users where a lot of them could have assumed different postures that strained the neck in the past year. In a study among a group of Dutch computer workers, neck and shoulder complaints were more frequently reported, even much more than the arm, the elbow and the hand [14]. The non computer users may not be straining their neck frequently but could have experienced musculoskeletal disorder on the neck since the assessment was during the past 12 months. Apart from computer use, other activities like driving, writing and reading could also have contributed to the high prevalence of neck symptoms.

We investigated whether some demographic characteristics and work factors were associated with SRMS among the computer workers. We found the risk of reporting musculoskeletal symptoms to be reducing in the older participants. The younger age represents the period where most individuals are more active in life and probably have more computer work load than the older workers in the same stations. Another possible reason for higher prevalence of musculoskeletal symptoms among younger computer users specifically could be because younger office workers use computers for longer periods than their senior counterparts, resulting in more musculoskeletal symptoms [15]. Our data shows that the highest duration of computer use among the computer users were found for the ages 30 years and below. In general, musculoskeletal symptoms are common among the middle-aged and people in active work life [4]. Janwantanakul et al [16] also claimed that office workers younger than 30 years were more likely to report musculoskeletal symptoms than those older than 49 years.

Our male computer users had higher prevalence of SRMS than the females and the females were less likely to report musculoskeletal symptoms. In contrast to this however, a previous study on computer users reported that experiencing pain in the neck or upper limb after work was more common among women than among men [17]. The prevalence of musculoskeletal symptoms in the head/neck, shoulders, upper back and ankles/feet was also reported to be higher in females than males [16]. The possible explanation for our somewhat varied findings could be that the males spent longer time on their computer work than the females or that the men were engaged in other activities that may precipitate or aggravate symptoms.

More musculoskeletal symptoms were likely to be reported with increase in daily time of computer use and in computer users with more years of computer use. This may be because the occurrence of musculoskeletal deviations and adaptations likely take place with sustained awkward positions over long periods of computer use. These findings corroborate previous studies. Daily computer usage longer than three hours was found to be significantly associated with the odds of reporting symptoms [18]. Kryger et al [3] had reported increased risk of new forearm pain to be associated with use of a mouse device for more than 30 hours per week, and with keyboard use more than 15 hours per week. Also according to Hakala et al [4], frequent computer-related activities are independent risk factors for neck-shoulder pain and low back pain. According to the authors, daily use of computers exceeding 2-3 hours seems to be a threshold for neck-shoulder pain and exceeding 5 hours for low back pain. Analyses of risk factors related to computer work have showed that for every quartile increase in weekly mouse usage, the risk for acute neck pain increased by 4% and the risk for acute shoulder pain by 10% [9]. Moderate evidence was also concluded for a positive association between the duration of mouse use and hand-arm symptoms [19]. Further, insufficient recovery after local muscle fatigue is also believed to be essential in the genesis of muscular pain in static work [20].

We found the computer tasks of data entry and designs/graphics to be associated with increased risk of reporting musculoskeletal symptoms among the computer users both over the past 7 days and during the past 12 months. This we postulate to be because these activities usually demand a higher concentration with the attendant possibility of the computer users inadvertently plunging into and sustaining awkward postures. In a study carried out in the United Kingdom to evaluate musculoskeletal disorders and visual strain in intensive data processing workers, 86% of data processors reported musculoskeletal pain/discomfort in the previous year, with the highest prevalence rate (58%) found for the neck [21]. This may be because computer work means sitting at desk with the neck in flexion position, while the keyboard and mouse operation requires repetitive upper extremity motions [4]. This posture and repetitive motions are likely to heighten musculoskeletal problems.

This study had a number of limitations. First, the outcome of this study may not necessarily confirm the actual presence and severity of the underlying musculoskeletal disorders in view of the fact that the study was a self reported cross sectional survey. However, the high prevalence of SRMS may point to the possibility of an underlying disorder. Our study was only able to account for the contribution of a few demographic confounders; however, SRMS may also be influenced by a number of other confounders that we were not able to account for in this study. This may include variables such as job stress, job motivation and satisfaction, individual pain tolerance, extracurricular activities and even psychological issues. According to Eltayeb et al [13], risk factors of musculoskeletal complaints in computer workers consist of a mixture of physical and psychosocial characteristics. Other confounders may relate to the computer itself such as different types of computer work stations, keyboards, mice and the actual computers that were being used. It was also difficult recruiting exclusive non computer users as controls for this study. This was because computer use has sufficiently gained popularity among most civil servants and recruiting and comparing individuals who never used computer at all with computer users will imply comparing individuals from different socioeconomic backgrounds, a move that may introduce severe bias. We however ensured that the non computer group were individuals with less than 30 minutes of weekly computer use. We also acknowledge that the estimation of duration of daily computer use may under or overestimate the contribution of duration of computer use to the observations made in this study.

In conclusion, computer users had significantly higher prevalence of SRMS over the past 7 days and during the last 12 month assessment durations. Over the past 7 days, the neck was mostly reported as the site of SRMS in the computer users while low back was the most prevalent for the non computer users. During the past 12 month period for the computer and non computer users, the neck was the site with most prevalent SRMS. Younger age, being male, working longer hours daily, increasing accumulated years of computer use, data entry tasks and computer designs/graphics were the significant risk factors for reporting musculoskeletal symptom among the computer users. Computer use may contribute to the high prevalence of SRMS among the computer users. For further studies, we recommend longitudinal studies of computer and non computer users that will be based on confirmatory tests for musculoskeletal disorders rather than self reports.

## Competing interests

The authors declare that they have no competing interests.

## Authors' contributions

OA was involved in the conceptualization, design, data collection and analysis and editing for intellectual content; BOOU involved in data collection, statistical analysis and interpretation and AFA interpreted data, involved in literature search and manuscript preparation. All the authors read and approved the final manuscript.

## Authors' information

OA is a Lecturer in the Department of Physiotherapy, University of Ibadan, Ibadan, Oyo State.

BOOU is a Clinical Physiotherapist at the Ladoke Akintola University of Technology, Osogbo, Osun State, Nigeria.

AFA is a Lecturer in the Department of Physiotherapy, University of Ibadan and Honorary Clinical Consultant, University College Hospital, Ibadan, Oyo State, Nigeria.

## Pre-publication history

The pre-publication history for this paper can be accessed here:

http://www.biomedcentral.com/1471-2474/11/177/prepub
